# High-Performance Sol–Gel-Derived CNT-ZnO Nanocomposite-Based Photodetectors with Controlled Surface Wrinkles

**DOI:** 10.3390/ma17215325

**Published:** 2024-10-31

**Authors:** Hee-Jin Kim, Seung Hun Lee, Dabin Jeon, Sung-Nam Lee

**Affiliations:** 1 Department of IT & Semiconductor Convergence Engineering, Tech University of Korea, Siheung 15073, Republic of Korea; 2 Department of Nano & Semiconductor Engineering, Tech University of Korea, Siheung 15073, Republic of Korea

**Keywords:** CNT, ZnO, sol–gel, photodetector

## Abstract

We investigate the effects of incorporating single-walled carbon nanotubes (CNTs) into sol–gel-derived ZnO thin films to enhance their optoelectronic properties for photodetector applications. ZnO thin films were fabricated on c-plane sapphire substrates with varying CNT concentrations ranging from 0 to 2.0 wt%. Characterization techniques, including high-resolution X-ray diffraction, photoluminescence, and atomic force microscopy, demonstrated the preferential growth of the ZnO (002) facet and improved optical properties with the increase in the CNT content. Electrical measurements revealed that the optimal CNT concentration of 1.5 wt% resulted in a significant increase in the dark current (from 0.34 mA to 1.7 mA) and peak photocurrent (502.9 µA), along with enhanced photoresponsivity. The rising and falling times of the photocurrent were notably reduced at this concentration, indicating improved charge dynamics due to the formation of a p-CNT/n-ZnO heterojunction. The findings suggest that the incorporation of CNTs not only modifies the structural and optical characteristics of ZnO thin films but also significantly enhances their electrical performance, positioning CNT-ZnO composites as promising candidates for advanced photodetector technologies in optoelectronic applications.

## 1. Introduction

Zinc oxide (ZnO) thin films have attracted significant attention as materials for light sensors, particularly due to their intrinsic optoelectronic properties [1,2]. ZnO has a wide direct bandgap of 3.37 eV and a high exciton binding energy of 60 meV, making it particularly well-suited for ultraviolet (UV) light detection [1,2,3]. These properties make ZnO an attractive material for applications where efficient detection and responsiveness to UV light are critical, such as environmental monitoring [3], UV imaging [4], and communication systems [5]. Moreover, the abundance of ZnO, combined with its chemical stability and non-toxic nature, adds to its appeal as a sustainable and cost-effective choice for photodetector applications [6,7]. ZnO thin films can be fabricated using various methods, such as sol–gel processing [7,8], sputtering [9], chemical vapor deposition [10], pulsed laser deposition [11], molecular beam epitaxy [12], and atomic layer deposition [13]. Among these, the sol–gel method is gaining popularity due to its low cost, simplicity, and versatility in forming various composite structures. The sol–gel process allows for precise control over film composition and morphology, making it suitable for scalable production [7,8]. Additionally, the sol–gel technique supports the formation of different types of thin films, including doped ZnO and composite structures, depending on the materials used in the solution [14,15]. This flexibility enables tailoring the optoelectronic properties of ZnO for specific applications.

Despite the advantages of ZnO as a material for light sensors, it has certain limitations in its pure form. ZnO is an n-type semiconductor due to intrinsic defects, such as zinc interstitials and oxygen vacancies [16,17]. These defects introduce free electrons into the conduction band, but the material still requires additional processes to enhance its conductivity and optimize its performance for photodetection. To address this, various strategies have been explored, including doping with other elements, creating nanostructures, and forming composite materials [14,15,18,19,20]. In particular, carbon-based nanomaterials, such as graphene, graphene oxide, and carbon nanotubes, are well-known for enhancing optoelectronic properties when combined with semiconductor films [21,22]. Among these, the integration of carbon nanotubes (CNTs) into ZnO thin films presents a promising approach for enhancing the performance of ZnO-based photodetectors [23,24]. CNTs, with their unique physical and chemical properties, have been widely applied in nanoelectronic devices, sensors, and catalysts [23,24,25,26,27]. They possess a high electrical and thermal conductivity, along with excellent chemical stability. Fractional descriptions have been investigated to improve the understanding of optoelectronic effects in CNTs [28]. These characteristics make CNTs an ideal candidate for improving the performance of ZnO-based light sensors [23]. Additionally, CNTs have a low density and small diameter, allowing for the formation of composite structures with ZnO without significantly increasing the weight or complexity of the device. Recently, ZnO-CNT composites have been developed by attaching ZnO nanoparticles to CNTs or incorporating CNTs onto ZnO thin films [29]. Incorporating CNTs into ZnO thin films can enhance charge transport, improve photocurrent generation, and accelerate response times in photodetectors [24]. In particular, single-walled CNTs (SWCNTs) have higher electrical conductivity than multi-walled CNTs (MWCNTs) due to better charge mobility and dispersing more uniformly in the ZnO matrix. Their smaller diameter and single-layer structure enable a better integration into the sol–gel solution, creating more homogeneous films. The high surface area of SWCNTs enhances interactions with ZnO, improving charge transfer and boosting the sensitivity and photoresponse of the photodetector. This is especially important for light sensors, where the speed and sensitivity of the device are critical performance metrics. One promising configuration for ZnO-based photodetectors is the metal–semiconductor–metal (MSM) structure. This structure offers several advantages, including simplicity in fabrication, high photocurrent gain, and enhanced responsivity. However, ZnO-based photodetectors fabricated via the sol–gel method may exhibit slow response times due to the polycrystalline nature of the ZnO films. The grain boundaries in polycrystalline ZnO can act as trap sites for charge carriers, limiting their mobility and slowing down the overall response of the device [30]. To mitigate this, significant research efforts have focused on improving the response time of ZnO-based photosensors through techniques such as nanostructuring, doping, and optimizing thin film growth conditions [31,32,33]. In recent years, the development of ZnO-CNT nanocomposite structures has emerged as a promising solution for overcoming the limitations of conventional ZnO photodetectors [23,34]. The incorporation of CNTs into the ZnO matrix can enhance charge carrier mobility and reduce recombination rates, resulting in faster response times and higher photodetection sensitivity. The high conductivity of CNTs helps improve charge transport, while their high surface area provides more active sites for light absorption and charge generation. Moreover, CNTs can extend the spectral response of ZnO photodetectors beyond the UV range, making them suitable for applications requiring broader wavelength detection [23,24]. In this study, we investigate the fabrication and performance of a high-efficiency photodetector based on a ZnO-CNT nanocomposite structure using the sol–gel method. This approach leverages the advantages of both ZnO and CNTs to create a photodetector with enhanced sensitivity, faster response times, and improved overall performance. By exploring the potential of ZnO-CNT composite materials, we aim to contribute to the advancement of next-generation optoelectronic devices capable of operating across a wide range of light wavelengths.

## 2. Materials and Methods

The fabrication of the CNT-ZnO-based light sensor was performed using the sol–gel deposition method, followed by the structural, optical, and electrical characterization of the nanocomposite. C-plane sapphire served as the substrate for ZnO thin-film growth. The sol–gel solution was prepared by mixing zinc acetate dihydrate and monoethanolamine at a 1:1 molar ratio. For the CNT-ZnO composite, SWCNTs (KORBON OS ET-D001, Gangneung, South Korea) were added to the ZnO sol solution at concentrations ranging from 0 wt% to 2.0 wt%. A separate CNT solution was prepared by dispersing 1.0 wt% SWCNTs in 2-methoxyethanol. The presence of SWCNTs in the film is clearly confirmed through Raman analysis, as evidenced by the distinct radial breathing mode (RBM) and the low D/G ratio, as shown in Figure S1a. The combined sol–gel solution was stirred at 1000 rpm for 30 min at 100 °C to ensure homogeneity. The ZnO-CNT solution was deposited onto the substrate using the spin coating technique. Five drops of the solution were applied, and spin coating was carried out at 6000 rpm for 30 s. After deposition, the film underwent a pre-bake at 200 °C for 5 min to remove excess solvent. This was followed by a post-bake at 900 °C for 1 min to improve the crystallinity of the ZnO thin film. In the CNT-ZnO composite films, no peaks related to CNTs were observed in the Raman spectra, as shown Figure S1b. This is likely due to the very small CNT content (<2.0 wt%) relative to the ZnO matrix, making it difficult to detect using Raman system. The CNT-ZnO-based light sensor was designed with a MSM configuration, with a sensing area of 1000 µm × 100 µm. The fabrication process involved a simple hard mask technique for patterning, and aluminum (Al) was deposited as the metal electrode with a thickness of 50 nm on the ZnO surface.

High-resolution X-ray diffraction (HR-XRD, (PANalytical, X’Pert Pro MRD, Tokyo, Japan)) with ω-2θ scanning was used to assess the crystallographic structure of the ZnO and CNT-ZnO composite films. Photoluminescence (PL) at 266 nm and UV-visible spectroscopy (ThermoFisher Scientific (Waltham, MA, USA), Evolution 300) were employed to evaluate the optical properties of the samples. The electrical conductivity of the films was determined using Hall effect measurements. Surface morphology was analyzed using atomic force microscopy (AFM, (NanoFocus, my−Scope plus, Seoul, Republic of Korea)) and scanning electron microscopy (SEM, (COXEM, EM-30, Daejeon, Republic of Korea)). The electrical properties of the M-S-M light sensor were measured using a semiconductor parameter analyzer. Current–voltage (I–V) measurements were performed under both dark conditions and UV illumination (365 nm, 5 mW). The photocurrent response over time was recorded to assess the sensor’s photo-reactivity. The photocurrent (ΔI_photo_) was calculated by subtracting the dark current (I_dark_) from the current under UV illumination (I_photo_).

## 3. Results and Discussion

**Figure 1:**
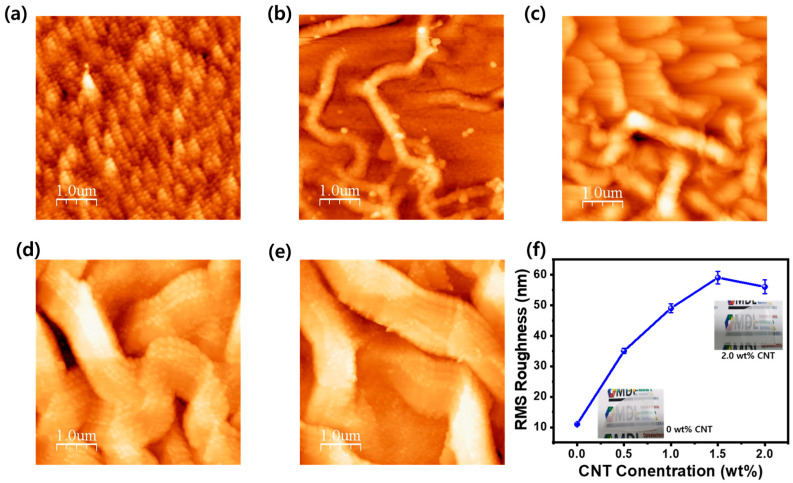
AFM images showing the surface morphology of ZnO thin films incorporated with CNTs at concentrations of (**a**) 0%, (**b**) 0.5 wt%, (**c**) 1.0 wt%, (**d**) 1.5 wt%, and (**e**) 2.0 wt%, fabricated using the sol–gel method. (**f**) RMS roughness of sol–gel-derived ZnO thin films embedded with varying concentrations of CNTs. Insets are photographic images of ZnO/sapphire and 2.0 wt% CNT-ZnO/sapphire.

Figure 1 presents AFM images of the surface morphology of ZnO thin films deposited using the sol–gel method, with increasing concentrations of CNTs (0 to 2.0 wt%). As shown in Figure 1a, the surface of the ZnO film without CNTs exhibits small, rounded grains aligned in a consistent direction. This orientation is due to the crystallographic isotropy of ZnO growth along the c-plane and is influenced by the rotational forces during the spin coating process. With the introduction of CNTs into the ZnO thin film, the surface morphology underwent significant changes. The structure transformed from small, grain-like particles to a more random, streaky appearance. This change can be attributed to the buckling effect induced by the CNTs. As CNTs are dispersed within the ZnO sol–gel solution, they act as mechanical stressors during the gel formation and subsequent crystallization process. Wrinkle formation in ZnO-CNT composite films occurs due to the thermal expansion mismatch between the ZnO matrix and CNTs during high-temperature treatment. As the ZnO matrix contracts during cooling, the CNTs resist this shrinkage, creating localized mechanical stress. This stress induces a buckling effect, leading to the characteristic wrinkled surface structure. The mismatch in thermal expansion coefficients between ZnO and CNTs, combined with the mechanical strain during the cooling process, plays a crucial role in this phenomenon [35]. The extent of this wrinkle formation increases with the CNT concentration, as demonstrated in Figure 1b–e. As more CNTs are incorporated, the mechanical stress within the film increases due to the larger number of CNTs interacting with the ZnO matrix. This interaction disrupts the uniform crystallization of ZnO, causing greater distortions in the surface structure. The CNTs, which have a high aspect ratio and stiffness, create localized areas of resistance against the ZnO matrix during drying and crystallization. This resistance amplifies the surface buckling effect, leading to a more pronounced wrinkle structure. In particular, the root mean square (RMS) surface roughness also increases as the CNT concentration rises. The roughness, which was measured at 11 nm for pure ZnO films, escalated to 35 nm at 0.5 wt% CNTs and further to 59 nm at 1.5 wt%. This increase in roughness is closely tied to the intensification of the buckling effect as more CNTs are added. Moreover, as the CNT content increases from 0.5 to 2.0 wt% in the CNT-ZnO composite films, the average wrinkle diameter significantly increases from 0.352 µm to 1.23 µm, respectively. This suggests a strong correlation between the CNT concentration and the size of the surface wrinkles. The wrinkled structure becomes more prominent as the CNTs, due to their superior mechanical strength and rigidity, further resist the shrinkage forces within the film during the post-bake process. This resistance to the contraction of the ZnO matrix not only causes a more pronounced wrinkling but also enhances the surface texture. However, as the CNT concentration reaches 2.0 wt%, the surface morphology does not exhibit further significant changes. This plateau in surface roughness is likely due to the saturation of CNTs within the ZnO matrix. At higher concentrations, the CNTs may begin to aggregate, reducing their ability to interact uniformly with the ZnO matrix. Additionally, as shown in Figure S2, the SEM analysis of large areas clearly reveals protrusions and depressions, indicating that regional differences in ZnO wrinkle thickness and CNT density may exist. 

The excessive CNT content could lead to a reduction in the mechanical interaction needed to amplify the buckling effect, as the ZnO matrix becomes too heavily influenced by the presence of CNTs to further increase surface distortions. Overall, the incorporation of CNTs into ZnO films leads to an increase in the wrinkle structure due to the buckling effect. This phenomenon is driven by the mechanical stress imposed by the CNTs during the thermal treatment of the sol–gel process, and it becomes more pronounced as the CNT concentration increases. Despite the formation of numerous surface wrinkle structures due to the incorporation of CNTs into ZnO, the overall macroscopic surface retains a mirror-like quality. As shown in the insets of Figure 1f, this surface is not only shiny but also semi-transparent, to the extent that the image behind it is clearly visible.

**Figure 2:**
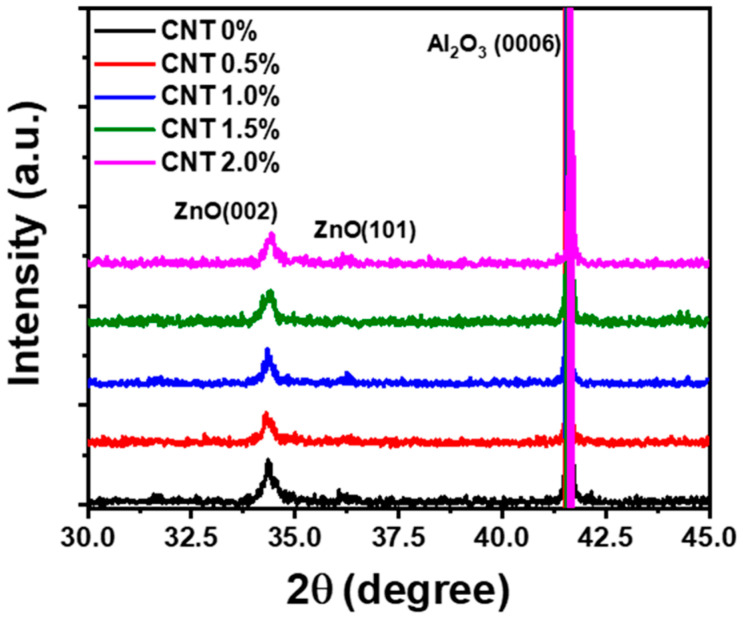
High-resolution X-ray diffraction (HR-XRD) patterns of ZnO thin films with varying CNT concentrations (0 to 2.0 wt%) deposited on c-plane sapphire substrates using the sol–gel method.

The crystallographic properties of CNT-incorporated ZnO thin films were analyzed using HR-XRD ω/2θ scans, as shown in Figure 2. The XRD patterns for ZnO films with varying CNT concentrations (0 to 2.0 wt%) were compared. Regardless of the CNT concentration, the results confirm that the (002) facet of ZnO remained the dominant crystal orientation, as indicated by the sharp peak near 34.4° corresponding to the ZnO (002) plane. This suggests that the ZnO films, grown using the sol–gel method on a c-plane sapphire substrate, exhibit strong c-axis orientation, which is the thermodynamically stable phase under the low- and high-temperature heat treatments. For the ZnO film without CNT incorporation (CNT 0%), a minor peak corresponding to the ZnO (101) plane around 36.3° was also observed, indicating the presence of a secondary orientation. However, as CNTs were incorporated into the films, the intensity of the ZnO (101) peak diminished slightly. This reduction in secondary crystallographic orientation suggests that CNT incorporation may disrupt the formation of additional planes, allowing the ZnO (002) plane to dominate. Additionally, it was observed that the (002) peak intensity slightly decreased, and the FWHM increased with the inclusion of CNTs. This broadening of the (002) peak, particularly at higher CNT concentrations, suggests a slight reduction in crystallinity. The CNTs likely introduce mechanical stress and strain during the high-temperature annealing process, disrupting the uniform growth of ZnO crystals. The buckling effect of CNTs, which is responsible for the surface wrinkle structure, may also contribute to this slight degradation in crystal orientation by inducing local distortions in the ZnO matrix during crystallization [35]. Despite these changes, the ZnO (002) phase remained well-defined even at CNT concentrations as high as 2.0 wt%. The data suggest that the incorporation of CNTs up to 2.0 wt% does not prevent the formation of high-quality ZnO (002) thin films, though the orientation and crystallinity are modestly affected by the presence of CNTs, likely due to mechanical interactions between the CNTs and the ZnO lattice during the sol–gel process.

**Figure 3:**
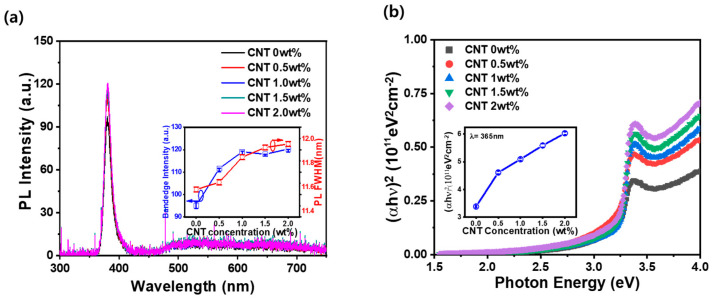
(**a**) Room temperature photoluminescence spectra of CNT-ZnO nanocomposite thin films excited with a 266 nm light source, and (**b**) absorbance spectra of CNT-ZnO nanocomposite thin films as a function of wavelength measured using ultraviolet–visible spectroscopy. Insets of (**a**,**b**) show the PL band-edge intensity, full width at half maximum (FWHM), and the (αhν)^2^ (10^11^ eV^2^ cm^−2^) values for sol–gel-derived ZnO thin films embedded with varying CNT concentrations, presented as a function of CNT incorporation.

**Figure 4:**
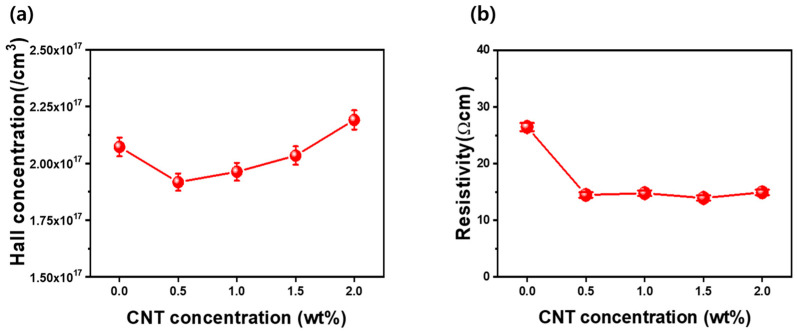
(**a**) Carrier concentration and (**b**) resistivity as a function of CNT concentration in CNT-ZnO nanocomposite thin films, measured using the van der Pauw method.

Figure 3a illustrates the optical properties of CNT-ZnO nanocomposite thin films, with PL measurements conducted using a 266 nm light source to analyze the effects of varying CNT concentrations. In the case of the ZnO thin film deposited via the sol–gel method without CNT incorporation, a strong PL band-edge emission was observed around 380 nm. This emission corresponds to the near-band-edge recombination of ZnO, demonstrating the high optical quality of the thin film [1]. Additionally, the film exhibited a relatively low deep-level emission beyond 450 nm, attributed to defects such as zinc interstitials and oxygen vacancies [16,17]. As the CNT concentration increased, the band-edge emission intensity also showed an upward trend. This suggests an enhancement in light extraction efficiency, likely due to the surface texturing effect induced by the CNTs, as observed in the previous surface morphology analysis (Figure 1). However, it is notable that while the band-edge emission increased, the deep-level emission remained relatively constant, irrespective of the CNT concentration. This phenomenon can be explained by the formation of a locally heterogeneous p-CNT/n-ZnO junction in the composite film. The introduction of CNTs into the ZnO matrix appears to increase carrier generation and recombination at the p-n heterojunctions, boosting the band-edge emission [36]. However, because the deep-level defects in ZnO are not significantly affected by this process, the corresponding deep-level luminescence does not show a similar increase. This selective enhancement in band-edge emission, without an increase in defect-related emissions, highlights the unique role of CNTs in improving the optical performance of the ZnO nanocomposite thin films. Figure 4b displays the Tauc plot, which is typically used to estimate the optical bandgap of materials. It shows the variation in (αhν)^2^ as a function of the photon energy for ZnO thin films with CNT concentrations ranging from 0 wt% to 2.0 wt%. For the pure ZnO film (0 wt% CNT), the bandgap appears to be around 3.2 eV, consistent with typical ZnO properties. As the CNT content increases, the absorption edge gradually shifts toward lower photon energies, indicating a potential reduction in the optical bandgap. This shift suggests improved light absorption, likely due to the introduction of additional energy states or enhanced charge separation at the CNT-ZnO interface. As a result, the absorption of light at a 365 nm wavelength increased progressively with increasing CNT incorporation from 0 to 2.0 wt%, as shown in the inset of Figure 4b. The incorporation of CNTs enhances the photon interaction with the ZnO matrix, creating more effective pathways for charge transfer. The gradual improvement in absorption efficiency aligns with the structural and electronic changes induced by the CNTs, enabling a more effective utilization of incoming light energy for charge generation.

Figure 4a,b present the carrier concentration and resistivity values of ZnO thin films as a function of CNT incorporation, measured using the Van der Pauw method. The carrier concentration of the ZnO thin film without CNT incorporation was determined to be 2.07 × 10^17^ cm^−3^. Upon incorporating CNTs at a concentration of 0.5 wt%, the carrier concentration decreased to 1.92 × 10^17^ cm^−3^. This initial reduction may be attributed to the disruption of the intrinsic n-type conductivity of ZnO due to the presence of CNTs, which could lead to charge carrier scattering or compensation effects. Specifically, the CNTs may introduce localized energy states that can trap electrons, thereby reducing the free carrier concentration in the ZnO matrix. As the CNT concentration increased to 1.0 wt%, the carrier concentration showed a notable increase to 2.0×10^17^ cm^−3^. This resurgence in carrier concentration suggests that, at this level of CNT incorporation, the beneficial effects of CNTs on electron mobility and charge transport begin to outweigh the initial scattering effects. The CNTs may facilitate enhanced electron transfer across the ZnO-CNT interface, improving overall conductivity. Furthermore, at a higher CNT concentration of 2.0 wt%, the carrier concentration increased further to 2.19 × 10^17^ cm^−3^. This increase can be attributed to a synergistic effect where the CNTs not only provide additional conductive pathways but also help maintain the structural integrity of the ZnO, allowing for a more favorable environment for electron transport. The presence of more CNTs could enhance electron delocalization, leading to an overall improvement in charge carrier density. Figure 4b illustrates the resistivity of ZnO thin films deposited by the sol–gel method as a function of the weight percentage of incorporated CNTs. The resistivity of the ZnO thin film without CNT incorporation was measured at 26.5 Ω-cm. Upon the addition of 0.5 wt% CNTs, a significant decrease in resistivity was observed, dropping to 14.5 Ω-cm. This reduction in resistivity can be attributed to enhanced charge carrier mobility facilitated by the CNTs, which provide conductive pathways that facilitate electron transport through the ZnO matrix.

**Figure 5:**
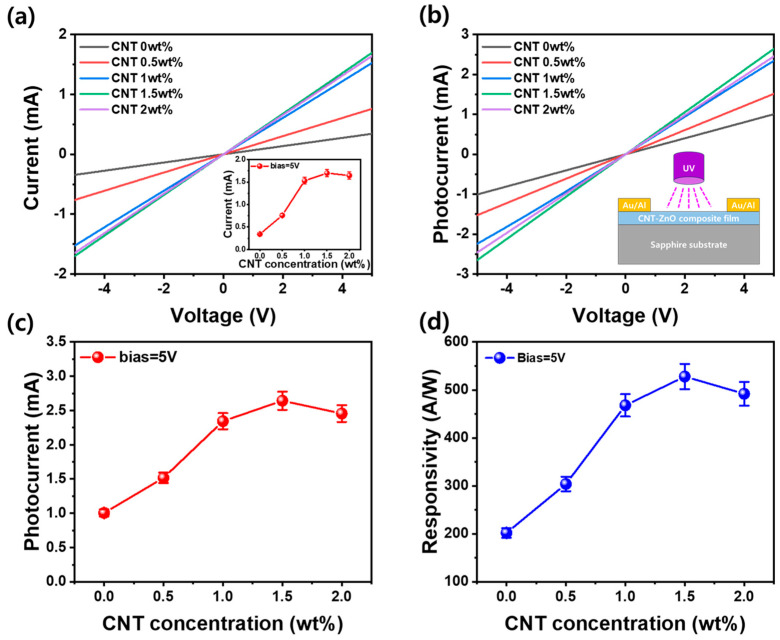
(**a**) Dark current–voltage and (**b**) photocurrent–voltage characteristics of the Al/ZnO-CNT/Al photodetector. The insets in (**a**,**b**) are a graph of the dark current as a function of CNT content at an applied voltage of 5.0 V and a schematic diagram of a CNT-ZnO nanocomposite photodetector with UV light applied, respectively. (**c**) Photocurrent and (**d**) photoresponsivity as a function of CNT content under a 5.0 V applied voltage.

Figure 5a presents a graph illustrating the voltage–dark current characteristics of a metal/ZnO-CNT/metal structure, where Al and Au metals are bonded to a ZnO film incorporating CNTs in concentrations ranging from 0 to 2.0 wt%. At an applied voltage of 5.0 V, the dark current for the ZnO film without CNTs was measured at 0.34 mA. However, this dark current increased significantly to 1.7 mA with the incorporation of CNTs up to 1.5 wt%, before slightly decreasing to 1.64 mA at 2.0 wt%. This trend can be attributed to the reduced resistivity of the ZnO film as CNTs were incorporated, which, as shown in Figure 4b, enhances the charge transport capabilities and results in an increased dark current. The increase in dark current with the addition of CNTs to the ZnO matrix is due to two main factors. First, CNTs provide a highly conductive pathway, reducing resistance and allowing electrons to travel more easily. This leads to higher current flow. Second, CNTs can introduce localized states in the bandgap of ZnO film, increasing trap density. As shown in Figure 4a, these additional trap states can lead to an increase in carrier concentration that can enhance non-radiative recombination, leading to an increase in dark current. The subsequent photocurrent versus applied voltage curve, obtained by subtracting the dark current from the UV current generated by applying 365 nm UV light to the metal/ZnO-CNT/metal structure photodetector, is shown in Figure 5b. At an applied voltage of 5.0 V, the photocurrent trends closely mirrored the behavior of the dark current. The photocurrent exhibited a gradual increase as the CNT concentration rose from 0 to 1.5 wt%, highlighting that the optimal incorporation of CNTs significantly enhances the photoreactivity of the device. Beyond this concentration, specifically at 2.0 wt%, the photocurrent diminished, as illustrated in Figure 5c, indicating that the photodetector’s efficiency is maximized at 1.5 wt%. Moreover, the photoresponsivity of photodetector also exhibited a similar trend, gradually increasing with CNT incorporation up to 1.5 wt% before decreasing with further increases, as shown in Figure 5d. The photoresponsivity (R_s_) was calculated using the formula of R_s_ = I_photo_ − I_dark_/P_0_A, where I_photo_ is the photocurrent, I_dark_ is the dark current, P_0_ represents the applied UV power, and A is the sensing area of the photodetector [37,38]. This emphasizes that both the dark current and photocurrent at 1.5 wt% are essential for achieving peak photoresponsivity in the CNT-ZnO MSM structure, thus optimizing its performance for practical applications in UV light detection. This optimal performance at 1.5 wt% can be further attributed to the adsorption and desorption processes of oxygen on the surface wrinkle structure of the ZnO-CNT nanocomposite when UV light is applied [38]. At this concentration, the presence of CNTs facilitates the formation of a favorable p-CNT/n-ZnO heterojunction, which enhances charge separation and improves the response to UV light. 

The UV illumination leads to the excitation of electrons in the ZnO, promoting the desorption of adsorbed oxygen molecules from the surface. The optimal balance of oxygen species enhances the formation of charge carriers, thus significantly increasing both the photocurrent and photoresponsivity. Moreover, the increase in CNT content contributes to the formation of surface wrinkle structures, as depicted in Figure 1, which can further enhance the distribution of active sites for photoreactions. As CNTs are incorporated, the surface wrinkle structure becomes more complex, creating additional paths for charge carriers and increasing the number of active sites for oxygen adsorption. The enhanced light intensity from the UV source interacts with these sites, facilitating photoreaction processes that can effectively eliminate oxygen. However, the excessive incorporation of CNTs beyond 1.5 wt% may lead to a saturation of these active sites, resulting in a decrease in both photocurrent and photoresponsivity. This indicates the importance of optimizing the CNT concentration to maximize both photoresponsivity and oxygen absorption capabilities in the ZnO-CNT photodetector system.

**Figure 6:**
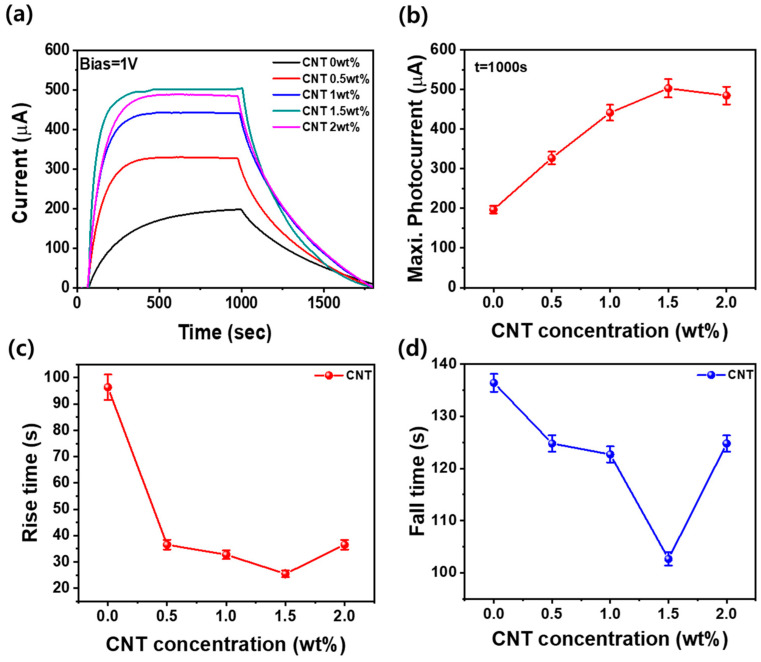
Photocurrent as a function of time after 950 s of excitation and termination of 365 nm ultraviolet light on an Al/ZnO-CNT/Al photodetector: (**a**) photocurrent response upon UV light application, (**b**) maximum photocurrent, (**c**) rise time, and (**d**) fall time after UV light termination as a function of the CNT content.

Figure 6a illustrates the photocurrent behavior over time following 950 s of UV excitation in the metal/CNT-ZnO/metal photodetector structure, along with the cessation of UV exposure. The maximum photocurrent exhibited a significant increase from 196.8 µA to 502.9 µA as the CNT content rose from 0 to 1.5 wt%. However, this maximum value slightly decreased to 484.7 µA when the CNT concentration was further increased to 2.0 wt%, as depicted in Figure 6b. The ZnO photodetector without CNTs exhibited a sluggish response to UV excitation, reaching its peak photocurrent after approximately 900 s. In contrast, the incorporation of CNTs resulted in a more rapid saturation of the photoexcited current compared to the pure ZnO photodetector. Notably, both the rising time—indicative of the photocurrent’s increase in response to UV light—and the falling time—reflecting the decrease in photocurrent after the UV light is turned off—were significantly shorter with CNT incorporation. The optimal performance was observed at a CNT content of 1.5 wt%, which resulted in the fastest rising and falling times, as illustrated in Figure 6c,d. This enhanced time-dependent reactivity at 1.5 wt% can be attributed to the formation of an optimal p-CNT/n-ZnO heterojunction, which facilitates efficient charge separation and transport. Additionally, the surface texture created by the CNTs improves the adsorption/desorption dynamics of oxygen species, enhancing the overall carrier dynamics in the photodetector.

## 4. Conclusions

We investigate the enhancement in sol–gel ZnO thin films through the incorporation of SWCNTs to develop effective photodetectors. Utilizing a C-plane sapphire substrate, ZnO thin films were fabricated with varying CNT concentrations. Characterization techniques, including HR-XRD and PL, revealed the preferential growth of the ZnO (002) facet and increased band-edge emission with CNT incorporation. The electrical properties were evaluated using Hall measurements, showing a decrease in resistivity and variations in carrier concentration with increasing CNT content. The optimal CNT concentration of 1.5 wt% resulted in a significant increase in the dark current (from 0.34 mA to 1.7 mA) and peak photocurrent (502.9 µA), along with improved photoresponsivity. The response time for photocurrent was notably faster at this concentration, indicating enhanced charge carrier dynamics facilitated by the formation of a p-CNT/n-ZnO heterojunction. Additionally, the study highlights the impact of surface morphology and the adsorption/desorption of oxygen species in the ZnO-CNT nanocomposite, contributing to the device’s improved performance. These findings suggest that CNTs significantly enhance the properties of ZnO-based photodetectors, making them promising for optoelectronic applications.

## Data Availability

The data presented in this study are available on request from the corresponding author. The data are not publicly available due to privacy concerns.

## Supplementary Materials

The following supporting information can be downloaded at: https://www.mdpi.com/article/10.3390/ma17215325/s1. Figure S1: (a) Raman spectrum of single-wall CNT films showing characteristic RBM, D, G, and G′-bands. (b) Raman spectra of CNT-ZnO composite films with varying CNT concentrations from 0 wt% to 2.0 wt%; Figure S2: SEM image of the wrinkle structure formation in CNT-ZnO composite film with 2.0 wt% CNTs.
