# Supplementary material for: High-Performance Sol–Gel-Derived CNT-ZnO Nanocomposite-Based Photodetectors with Controlled Surface Wrinkles
Source: Materials (Basel). 2024 Oct 31;17(21):5325. doi: 10.3390/ma17215325 (PMC11547469; doi:10.3390/ma17215325)
Supplement: Supplementary file 1 [file materials-17-05325-s001.zip › materials-3276733-supplementary.pdf]

Supplemental Information

High-Performance Sol–Gel-Derived CNT-ZnO Nanocomposite-Based Photodetectors with Controlled Surface Wrinkles

Hee-Jin Kim [1], Seung Hoon Lee [1], Dabin Jeon [1] and Sung-Nam Lee [1,2,*]

[1] Department of IT & Semiconductor Convergence Engineering, Tech University of Korea,
Siheung 15073, Republic of Korea
[2] Department of Nano & Semiconductor Engineering, Tech University of Korea,
Siheung 15073, Republic of Korea
* Correspondence: snlee@tukorea.ac.kr; Tel.: +82-31-8041-0721

S1. Raman analysis of single walled CNT films and CNT-ZnO composite films

S2. Large area analysis of surface wrinkle structure in CNT-ZnO composite films

S1. Raman analysis of single walled CNT film and CNT-ZnO composite films with different CNT concentrations

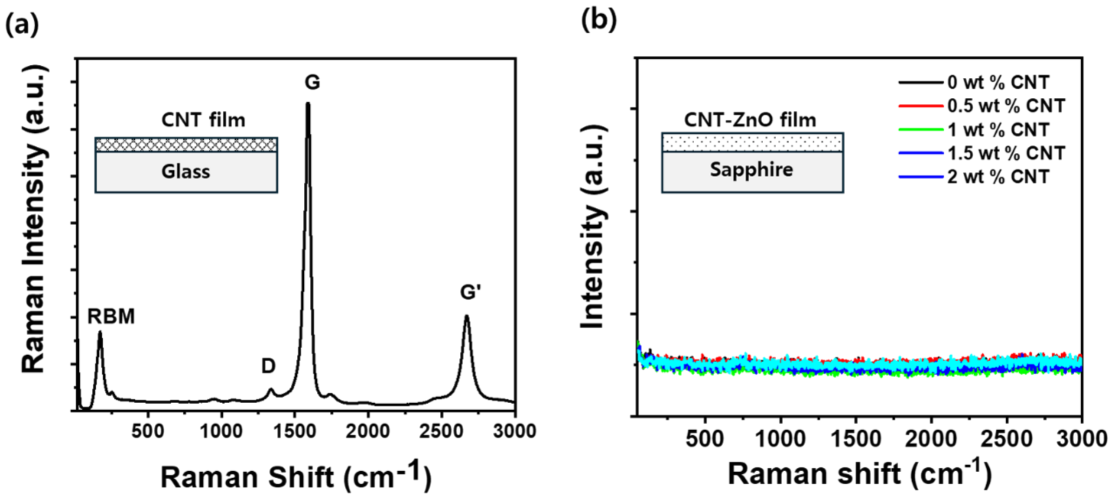

Figure S1. (a) Raman spectrum of single-wall CNT films showing characteristic RBM, D, G, and G'-bands. (b) Raman spectra of CNT-ZnO composite films with varying CNT concentrations from 0 wt% to 2.0 wt%.

Figure S1 shows the Raman spectra show characteristic peaks for RBM, D, G, and G' modes. The RBM peak at 170 cm^{-1}, a hallmark of single-walled CNTs (SWCNTs), confirms their presence. Additionally, the D/G intensity ratio was very low due to a strong G peak at 1580 cm^{-1} and a relatively weak D peak at 1350 cm^{-1}. This low D/G ratio indicates minimal structural defects, further confirming the use of SWCNTs rather than multi-walled CNTs (MWCNTs), which typically have a higher D-band to G-band ratio. However, in the CNT-ZnO composite films, no peaks related to CNTs were observed in the Raman spectra, as shown Fig. S1(b). This is likely due to the very small CNT content (< 2.0 wt%) relative to the ZnO matrix, making it difficult to detect using our Raman system. Additionally, Raman analysis of the CNT films (Fig. S1(a)) shows minimal defect like amorphous carbon. Assuming no significant increase during the formation of CNT-ZnO composite films, it is reasonable to expect that the amorphous carbon content in these composite films is similarly low. This suggests that amorphous carbon will have a negligible effect on the overall structure and performance of the composite.

S2. Large area analysis of surface wrinkle structure in CNT-ZnO composite films

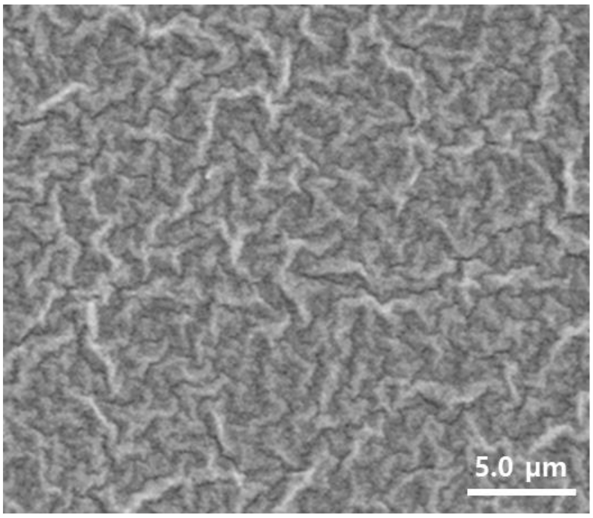

Figure S2. SEM image of wrinkle structure formation in CNT-ZnO composite film with 2.0 wt% CNTs

Figure S2 presents a SEM image showing the wrinkle-like surface structure of ZnO with a 2 wt% CNT concentration over a large area. The image demonstrates that the relatively flat surface transforms into a wrinkled texture, featuring randomly distributed protrusions and depressions. This change in surface morphology suggests that the incorporation of CNTs leads to the development of a complex surface structure, which is consistent across a broader area. Notably, the protrusions and depressions were distinctly observed, suggesting that regional variations in the ZnO wrinkle thickness and CNT density may occur due to shrinkage during the cooling phase following thermal treatment
